# 3-(Adamantan-1-yl)-4-[(*E*)-(2,6-di­fluoro­benzyl­idene)amino]-1-[(4-ethyl­piperazin-1-yl)meth­yl]-4,5-di­hydro-1*H*-1,2,4-triazole-5-thione

**DOI:** 10.1107/S1600536813009264

**Published:** 2013-04-10

**Authors:** Abdul-Malek S. Al-Tamimi, Ebtehal S. Al-Abdullah, Ali A. El-Emam, Seik Weng Ng, Edward R. T. Tiekink

**Affiliations:** a Department of Pharmaceutical Chemistry, College of Pharmacy, Salman bin Abdulaziz University, Alkharj 11942, Saudi Arabia; b Department of Pharmaceutical Chemistry, College of Pharmacy, King Saud University, Riyadh 11451, Saudi Arabia; c Department of Chemistry, University of Malaya, 50603 Kuala Lumpur, Malaysia; d Chemistry Department, Faculty of Science, King Abdulaziz University, PO Box 80203 Jeddah, Saudi Arabia

## Abstract

In the title compound, C_26_H_34_F_2_N_6_S, the triazole ring is linked to a benzene ring *via* an imine bond [N=C = 1.255 (2) Å; conformation: *E*], with a dihedral angle of 25.21 (11)° between the rings. The 4-ethyl­piperazinyl residue is folded away from the thione-S atom. In the crystal, helical supra­molecular chains propagating along [010] and sustained by weak C—S⋯π(triazole) inter­actions occur [S⋯centroid distance = 3.2872 (10) Å]. Links between these chains are of the type benzene-C—H⋯N(imine) and π–π [between centrosymmetrically related benzene rings with an inter-centroid distance of 3.9241 (15) Å] and result in a three-dimensional architecture.

## Related literature

For background to the pharmacological properties of adamantane derivatives, see: Al-Omar *et al.* (2010 ▶). For related structures, see: Almutairi *et al.* (2012 ▶); El-Emam *et al.* (2012 ▶).
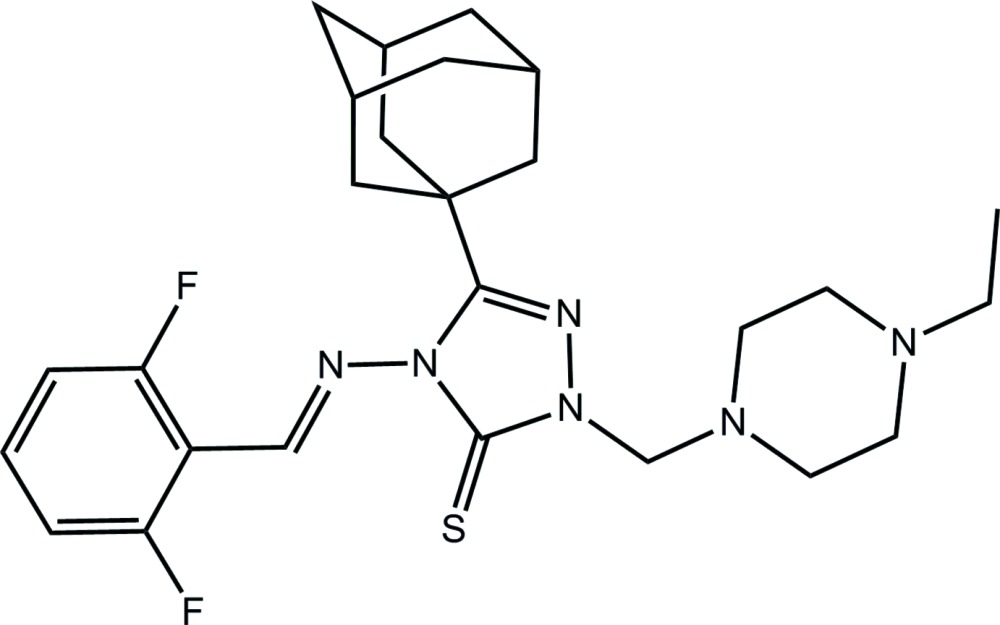


## Experimental

### 

#### Crystal data

C_26_H_34_F_2_N_6_S
*M* *_r_* = 500.65
Monoclinic, 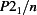

*a* = 17.0824 (11) Å
*b* = 7.8212 (6) Å
*c* = 19.6691 (14) Å
β = 92.249 (6)°
*V* = 2625.9 (3) Å^3^
*Z* = 4
Mo *K*α radiation
μ = 0.16 mm^−1^
*T* = 295 K
0.40 × 0.30 × 0.10 mm

#### Data collection

Agilent SuperNova Dual diffractometer with Atlas detector
Absorption correction: multi-scan (*CrysAlis PRO*; Agilent, 2011 ▶) *T*_min_ = 0.656, *T*_max_ = 1.000
16764 measured reflections
6063 independent reflections
3908 reflections with *I* > 2σ(*I*)
*R*_int_ = 0.038

#### Refinement

*R*[*F* ^2^ > 2σ(*F* ^2^)] = 0.053
*wR*(*F* ^2^) = 0.138
*S* = 1.01
6063 reflections
316 parameters
H-atom parameters constrained
Δρ_max_ = 0.20 e Å^−3^
Δρ_min_ = −0.25 e Å^−3^

### 

Data collection: *CrysAlis PRO* (Agilent, 2011 ▶); cell refinement: *CrysAlis PRO*; data reduction: *CrysAlis PRO*; program(s) used to solve structure: *SHELXS97* (Sheldrick, 2008 ▶); program(s) used to refine structure: *SHELXL97* (Sheldrick, 2008 ▶); molecular graphics: *ORTEP-3 for Windows* (Farrugia, 2012 ▶) and *DIAMOND* (Brandenburg, 2006 ▶); software used to prepare material for publication: *publCIF* (Westrip, 2010 ▶).

## Supplementary Material

Crystal structure: contains datablock(s) global, I. DOI: 10.1107/S1600536813009264/hb7065sup1.cif

Structure factors: contains datablock(s) I. DOI: 10.1107/S1600536813009264/hb7065Isup2.hkl

Supplementary material file. DOI: 10.1107/S1600536813009264/hb7065Isup3.cml

Additional supplementary materials:  crystallographic information; 3D view; checkCIF report

## Figures and Tables

**Table 1:** Hydrogen-bond geometry (Å, °) *Cg*1 is the centroid of the N1–N3,C1,C2 ring

*D*—H⋯*A*	*D*—H	H⋯*A*	*D*⋯*A*	*D*—H⋯*A*
C18—H18*A*⋯N5^i^	0.93	2.58	3.451 (3)	157
C1—S1⋯*Cg*1^ii^	1.66 (1)	3.29 (1)	4.849 (2)	155 (1)

Symmetry codes: (i) 
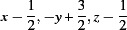
; (ii) 
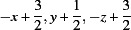
.

**Fig. 1.**
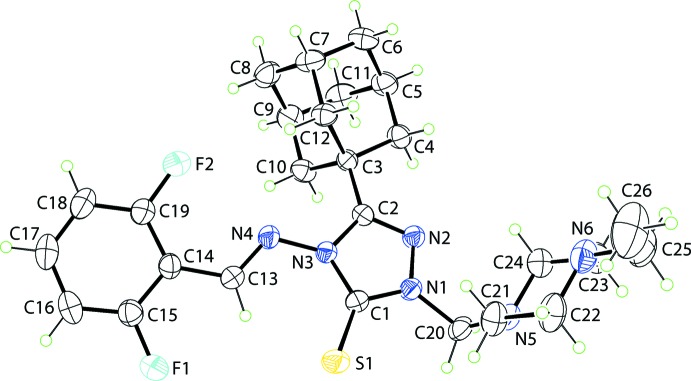
The molecular structure of (I) showing displacement ellipsoids at the 35% probability level.

**Fig. 2.**
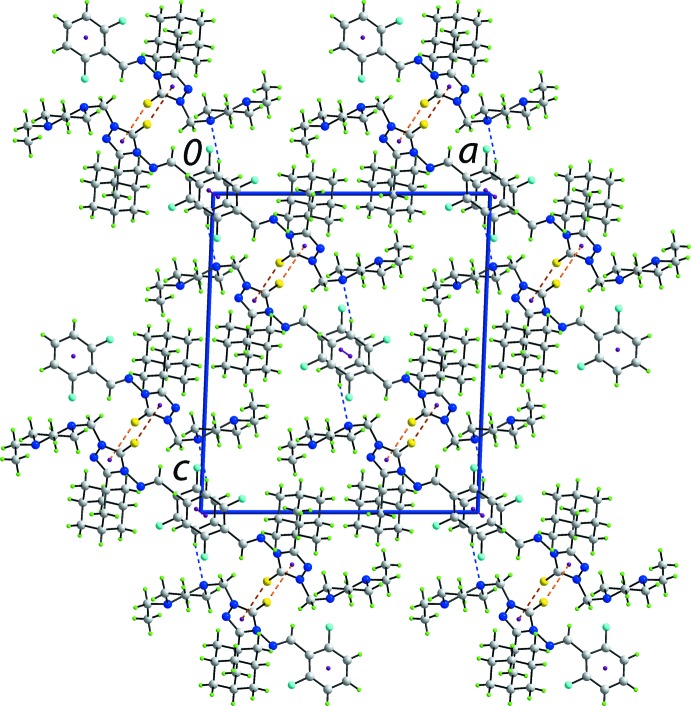
A view of the unit-cell contents in projection down the b axis of (I). The C—H···N, C—S···π and π—π interactions are shown as blue, orange and purple dashed lines, respectively.

## supplementary crystallographic information

### Comment

The title compound, (I), was synthesized as a potential chemotherapeutic agent (Al-Omar *et al.*, 2010) in continuation of our interest in the chemical and pharmacological properties of adamantane derivatives and allied structural studies (El-Emam *et al.*, 2012; Almutairi *et al.*, 2012).

In (I), Fig. 1, the triazole ring is almost planar (r.m.s. deviation of the fitted atoms = 0.009 Å) and is linked to an inclined benzene ring via an imine bond [N4═C13 = 1.255 (2) Å; *E* configuration] forming a dihedral angle of 25.21 (11)°. The twist occurs between the triazole ring and the imine bond as seen in the value of the C1—N3—N4—C13 torsion angle of -28.1 (3)°. The piperazinyl residue (chair conformation) is folded away from the thione-S1 atom.

Helical supramolecular chains along the *b* axis direction and sustained by C—S···π(triazole) interactions feature in the crystal structure. These are connected into a three-dimensional architecture by benzene-*C*—*H*···*N*(imine), Table 1, and π—π interactions between centrosymmetrically related benzene rings [inter-centroid distance = 3.9241 (15) Å for symmetry operation: 1 - *x*, 2 - *y*, 1 - *z*], Fig. 2.

### Experimental

A mixture of the 5-(adamantan-1-yl)-4-(2,6-difluorobenzylideneamino)-4*H*-1,2,4-triazole-3-thiol (347 mg, 1 mmol), 1-ethylpiperazine (114 mg, 1 mmol) and 37% formaldehyde solution (0.5 ml), in ethanol (8 ml), was heated under reflux for 15 min. when a clear solution was obtained. Stirring was continued for 12 h at room temperature and the mixture was allowed to stand overnight. Cold water (5 ml) was added and the reaction mixture was stirred for 20 min. The precipitated crude product was filtered, washed with water, dried, and crystallized from ethanol to yield 445 mg (89%) of the title compound (C_26_H_34_F_2_N_6_S) as crystals. *M*.pt: 442–444 K. Yellow prisms were obtained by slow evaporation of its CHCl_3_:EtOH (1:1; 5 ml) solution at room temperature. ^1^H NMR (CDCl_3_, 500.13 MHz): δ 1.07 (t, 3H, CH_3_, J = 7.0 Hz), 1.78 (s, 6H, adamantane-H), 2.08 (s, 3H, adamantane-H), 2.16 (s, 6H, adamantane-H), 2.40 (q, 2H, C*H*_2_CH_3_, J = 7.0 Hz), 2.48–2.49 (m, 4H, piperazine-H), 2.92 (s, 4H, piperazine-H), 5.18 (s, 2H, CH_2_), 7.02 (t, 2H, Ar—H, J = 8.5 Hz), 7.44–7.48 (m, 1H, Ar—H), 10.62 (s, 1H, CH=N). ^13^C NMR (CDCl_3_, 125.76 MHz): δ 11.91 (CH_3_), 28.0, 35.50, 36.46, 38.33 (adamantane-C), 50.42, 52.80 (piperazine-C), 52.32 (*C*H_2_CH_3_), 68.79 (CH_2_), 110.89, 112.16, 133.20, 152.22 (Ar—C), 155.45, 161.0 (triazole C-5 & CH=N), 163.14 (C=S).

### Refinement

The H-atoms were placed in calculated positions [and C—H = 0.93 to 0.98 Å, *U*_iso_(H) = 1.2–1.5*U*_eq_(C)] and were included in the refinement in the riding model approximation.

### Crystal data

**Table:**

C_26_H_34_F_2_N_6_S	*F*(000) = 1064
*M**_r_* = 500.65	*D*_x_ = 1.266 Mg m^−^^3^
Monoclinic, *P*2_1_/*n*	Mo *K*α radiation, λ = 0.71073 Å
Hall symbol: -P 2yn	Cell parameters from 3864 reflections
*a* = 17.0824 (11) Å	θ = 2.9–27.5°
*b* = 7.8212 (6) Å	µ = 0.16 mm^−^^1^
*c* = 19.6691 (14) Å	*T* = 295 K
β = 92.249 (6)°	Prism, yellow
*V* = 2625.9 (3) Å^3^	0.40 × 0.30 × 0.10 mm
*Z* = 4	

### Data collection

**Table:**

Agilent SuperNova Dual diffractometer with Atlas detector	6063 independent reflections
Radiation source: SuperNova (Mo) X-ray Source	3908 reflections with *I* > 2σ(*I*)
Mirror monochromator	*R*_int_ = 0.038
Detector resolution: 10.4041 pixels mm^-1^	θ_max_ = 27.6°, θ_min_ = 2.9°
ω scan	*h* = −22→20
Absorption correction: multi-scan (*CrysAlis PRO*; Agilent, 2011)	*k* = −9→10
*T*_min_ = 0.656, *T*_max_ = 1.000	*l* = −25→25
16764 measured reflections	

### Refinement

**Table:**

Refinement on *F*^2^	Primary atom site location: structure-invariant direct methods
Least-squares matrix: full	Secondary atom site location: difference Fourier map
*R*[*F*^2^ > 2σ(*F*^2^)] = 0.053	Hydrogen site location: inferred from neighbouring sites
*wR*(*F*^2^) = 0.138	H-atom parameters constrained
*S* = 1.01	*w* = 1/[σ^2^(*F*_o_^2^) + (0.0532*P*)^2^ + 0.6004*P*] where *P* = (*F*_o_^2^ + 2*F*_c_^2^)/3
6063 reflections	(Δ/σ)_max_ < 0.001
316 parameters	Δρ_max_ = 0.20 e Å^−^^3^
0 restraints	Δρ_min_ = −0.25 e Å^−^^3^

### Special details

**Table:**

Geometry. All e.s.d.'s (except the e.s.d. in the dihedral angle between two l.s. planes) are estimated using the full covariance matrix. The cell e.s.d.'s are taken into account individually in the estimation of e.s.d.'s in distances, angles and torsion angles; correlations between e.s.d.'s in cell parameters are only used when they are defined by crystal symmetry. An approximate (isotropic) treatment of cell e.s.d.'s is used for estimating e.s.d.'s involving l.s. planes.
Refinement. Refinement of *F*^2^ against ALL reflections. The weighted *R*-factor *wR* and goodness of fit *S* are based on *F*^2^, conventional *R*-factors *R* are based on *F*, with *F* set to zero for negative *F*^2^. The threshold expression of *F*^2^ > σ(*F*^2^) is used only for calculating *R*-factors(gt) *etc*. and is not relevant to the choice of reflections for refinement. *R*-factors based on *F*^2^ are statistically about twice as large as those based on *F*, and *R*- factors based on ALL data will be even larger.

### Fractional atomic coordinates and isotropic or equivalent isotropic displacement parameters (Å^2^)

**Table:**

	*x*	*y*	*z*	*U*_iso_*/*U*_eq_	
S1	0.74938 (3)	0.92905 (8)	0.71719 (3)	0.05854 (19)	
F1	0.51929 (7)	0.8699 (2)	0.63956 (7)	0.0759 (4)	
F2	0.64752 (8)	0.6197 (2)	0.45778 (7)	0.0901 (5)	
N1	0.86739 (9)	0.6995 (2)	0.71058 (7)	0.0417 (4)	
N2	0.89390 (8)	0.5649 (2)	0.67246 (8)	0.0419 (4)	
N3	0.78219 (8)	0.6639 (2)	0.62927 (7)	0.0382 (4)	
N4	0.72072 (9)	0.6716 (2)	0.58085 (8)	0.0447 (4)	
N5	0.97927 (9)	0.8412 (2)	0.77067 (8)	0.0483 (4)	
N6	1.10661 (11)	1.0150 (3)	0.71147 (10)	0.0668 (6)	
C1	0.79839 (10)	0.7645 (3)	0.68619 (9)	0.0408 (4)	
C2	0.84230 (10)	0.5462 (2)	0.62267 (9)	0.0370 (4)	
C3	0.84653 (10)	0.4095 (2)	0.56948 (9)	0.0382 (4)	
C4	0.92160 (11)	0.3045 (3)	0.58368 (11)	0.0501 (5)	
H4A	0.9670	0.3785	0.5816	0.060*	
H4B	0.9209	0.2559	0.6290	0.060*	
C5	0.92751 (13)	0.1613 (3)	0.53147 (11)	0.0568 (6)	
H5A	0.9755	0.0957	0.5413	0.068*	
C6	0.93019 (13)	0.2356 (3)	0.46007 (12)	0.0627 (6)	
H6A	0.9342	0.1441	0.4271	0.075*	
H6B	0.9757	0.3090	0.4568	0.075*	
C7	0.85525 (13)	0.3389 (3)	0.44517 (11)	0.0576 (6)	
H7A	0.8566	0.3872	0.3993	0.069*	
C8	0.78406 (14)	0.2228 (3)	0.44952 (12)	0.0661 (7)	
H8A	0.7868	0.1323	0.4160	0.079*	
H8B	0.7366	0.2881	0.4399	0.079*	
C9	0.78190 (13)	0.1457 (3)	0.52052 (12)	0.0594 (6)	
H9A	0.7364	0.0698	0.5229	0.071*	
C10	0.77556 (11)	0.2883 (3)	0.57310 (11)	0.0502 (5)	
H10A	0.7739	0.2391	0.6183	0.060*	
H10B	0.7275	0.3520	0.5643	0.060*	
C11	0.85704 (14)	0.0427 (3)	0.53560 (13)	0.0660 (6)	
H11A	0.8558	−0.0072	0.5807	0.079*	
H11B	0.8610	−0.0492	0.5028	0.079*	
C12	0.84933 (12)	0.4845 (3)	0.49680 (9)	0.0466 (5)	
H12A	0.8024	0.5510	0.4867	0.056*	
H12B	0.8943	0.5597	0.4939	0.056*	
C13	0.65661 (11)	0.7314 (3)	0.59892 (10)	0.0502 (5)	
H13A	0.6525	0.7697	0.6434	0.060*	
C14	0.58792 (11)	0.7427 (3)	0.55192 (10)	0.0464 (5)	
C15	0.51815 (12)	0.8099 (3)	0.57474 (11)	0.0533 (5)	
C16	0.44989 (12)	0.8209 (3)	0.53597 (14)	0.0657 (7)	
H16A	0.4048	0.8667	0.5538	0.079*	
C17	0.44969 (14)	0.7628 (4)	0.47009 (14)	0.0707 (7)	
H17A	0.4039	0.7686	0.4430	0.085*	
C18	0.51624 (14)	0.6963 (3)	0.44379 (12)	0.0681 (7)	
H18A	0.5162	0.6582	0.3990	0.082*	
C19	0.58297 (12)	0.6868 (3)	0.48466 (11)	0.0575 (6)	
C20	0.90864 (11)	0.7436 (3)	0.77544 (9)	0.0502 (5)	
H20A	0.8729	0.8074	0.8029	0.060*	
H20B	0.9213	0.6382	0.7995	0.060*	
C21	0.96781 (13)	1.0051 (3)	0.73711 (14)	0.0649 (6)	
H21A	0.9265	1.0680	0.7586	0.078*	
H21B	0.9519	0.9872	0.6898	0.078*	
C22	1.04327 (14)	1.1080 (3)	0.74151 (15)	0.0741 (7)	
H22A	1.0354	1.2160	0.7179	0.089*	
H22B	1.0571	1.1326	0.7888	0.089*	
C23	1.11758 (13)	0.8541 (4)	0.74629 (13)	0.0683 (7)	
H23A	1.1315	0.8749	0.7939	0.082*	
H23B	1.1602	0.7918	0.7266	0.082*	
C24	1.04390 (12)	0.7486 (3)	0.74069 (12)	0.0600 (6)	
H24A	1.0309	0.7244	0.6932	0.072*	
H24B	1.0521	0.6407	0.7642	0.072*	
C25	1.17960 (16)	1.1158 (4)	0.71267 (15)	0.0885 (9)	
H25A	1.2226	1.0420	0.7008	0.106*	
H25B	1.1905	1.1577	0.7585	0.106*	
C26	1.1755 (2)	1.2638 (5)	0.6647 (2)	0.1292 (15)	
H26A	1.2245	1.3238	0.6667	0.194*	
H26B	1.1343	1.3398	0.6774	0.194*	
H26C	1.1648	1.2231	0.6193	0.194*	

### Atomic displacement parameters (Å^2^)

**Table:**

	*U*^11^	*U*^22^	*U*^33^	*U*^12^	*U*^13^	*U*^23^
S1	0.0632 (3)	0.0571 (4)	0.0552 (3)	0.0145 (3)	0.0015 (3)	−0.0143 (3)
F1	0.0556 (7)	0.0991 (12)	0.0733 (9)	0.0090 (7)	0.0078 (6)	−0.0235 (8)
F2	0.0756 (9)	0.1407 (15)	0.0533 (8)	0.0358 (9)	−0.0064 (6)	−0.0250 (9)
N1	0.0439 (8)	0.0442 (10)	0.0368 (8)	0.0010 (7)	−0.0019 (6)	−0.0016 (8)
N2	0.0426 (8)	0.0421 (10)	0.0410 (8)	0.0024 (7)	−0.0006 (7)	−0.0006 (8)
N3	0.0387 (8)	0.0404 (9)	0.0354 (8)	0.0036 (7)	−0.0007 (6)	−0.0004 (7)
N4	0.0427 (9)	0.0483 (11)	0.0426 (9)	0.0081 (8)	−0.0062 (7)	−0.0041 (8)
N5	0.0498 (9)	0.0526 (11)	0.0417 (9)	−0.0019 (8)	−0.0072 (7)	0.0020 (8)
N6	0.0611 (12)	0.0748 (15)	0.0635 (12)	−0.0167 (11)	−0.0089 (9)	0.0042 (11)
C1	0.0444 (10)	0.0418 (11)	0.0362 (9)	−0.0007 (9)	0.0023 (8)	−0.0001 (9)
C2	0.0373 (9)	0.0354 (11)	0.0384 (9)	0.0020 (8)	0.0035 (7)	0.0034 (8)
C3	0.0381 (9)	0.0368 (11)	0.0398 (10)	0.0030 (8)	0.0014 (7)	−0.0004 (9)
C4	0.0517 (11)	0.0468 (13)	0.0517 (12)	0.0105 (10)	0.0013 (9)	0.0010 (10)
C5	0.0631 (13)	0.0478 (14)	0.0599 (13)	0.0173 (11)	0.0045 (10)	−0.0031 (11)
C6	0.0699 (14)	0.0596 (16)	0.0600 (14)	0.0107 (12)	0.0193 (11)	−0.0089 (12)
C7	0.0789 (15)	0.0544 (14)	0.0401 (11)	0.0126 (12)	0.0087 (10)	0.0014 (10)
C8	0.0753 (15)	0.0613 (16)	0.0606 (14)	0.0078 (13)	−0.0098 (11)	−0.0209 (13)
C9	0.0603 (13)	0.0469 (14)	0.0712 (15)	−0.0106 (11)	0.0064 (11)	−0.0105 (12)
C10	0.0508 (11)	0.0447 (13)	0.0559 (12)	−0.0036 (10)	0.0114 (9)	−0.0031 (10)
C11	0.0965 (18)	0.0390 (13)	0.0629 (14)	0.0051 (12)	0.0092 (13)	−0.0024 (12)
C12	0.0545 (11)	0.0438 (12)	0.0420 (10)	0.0049 (9)	0.0067 (8)	0.0045 (9)
C13	0.0485 (11)	0.0614 (14)	0.0404 (10)	0.0051 (10)	−0.0012 (8)	−0.0058 (10)
C14	0.0428 (10)	0.0470 (13)	0.0488 (11)	0.0028 (9)	−0.0039 (8)	0.0010 (10)
C15	0.0485 (12)	0.0530 (14)	0.0582 (13)	−0.0008 (10)	0.0000 (9)	−0.0024 (11)
C16	0.0401 (11)	0.0701 (17)	0.0865 (18)	0.0023 (11)	−0.0024 (11)	0.0072 (15)
C17	0.0515 (14)	0.0808 (19)	0.0779 (17)	−0.0066 (13)	−0.0202 (12)	0.0120 (15)
C18	0.0685 (15)	0.0814 (18)	0.0531 (13)	−0.0028 (14)	−0.0168 (11)	0.0013 (13)
C19	0.0555 (12)	0.0675 (16)	0.0489 (12)	0.0103 (12)	−0.0052 (10)	−0.0026 (12)
C20	0.0562 (12)	0.0604 (14)	0.0335 (10)	−0.0021 (11)	−0.0046 (8)	0.0004 (10)
C21	0.0625 (14)	0.0539 (15)	0.0767 (16)	−0.0021 (12)	−0.0164 (11)	0.0048 (13)
C22	0.0722 (16)	0.0556 (16)	0.0923 (19)	−0.0100 (13)	−0.0243 (14)	0.0042 (15)
C23	0.0545 (13)	0.0802 (19)	0.0695 (16)	−0.0003 (13)	−0.0037 (11)	0.0011 (14)
C24	0.0553 (13)	0.0628 (16)	0.0616 (14)	0.0002 (11)	−0.0027 (10)	0.0017 (12)
C25	0.0774 (17)	0.105 (2)	0.0817 (19)	−0.0353 (17)	−0.0109 (14)	0.0082 (18)
C26	0.125 (3)	0.132 (3)	0.128 (3)	−0.066 (2)	−0.029 (2)	0.055 (3)

### Geometric parameters (Å, º)

**Table:**

S1—C1	1.664 (2)	C9—C11	1.534 (3)
F1—C15	1.358 (2)	C9—H9A	0.9800
F2—C19	1.348 (2)	C10—H10A	0.9700
N1—C1	1.354 (2)	C10—H10B	0.9700
N1—N2	1.379 (2)	C11—H11A	0.9700
N1—C20	1.474 (2)	C11—H11B	0.9700
N2—C2	1.300 (2)	C12—H12A	0.9700
N3—C1	1.387 (2)	C12—H12B	0.9700
N3—C2	1.389 (2)	C13—C14	1.468 (3)
N3—N4	1.391 (2)	C13—H13A	0.9300
N4—C13	1.255 (2)	C14—C19	1.393 (3)
N5—C20	1.434 (3)	C14—C15	1.393 (3)
N5—C21	1.452 (3)	C15—C16	1.371 (3)
N5—C24	1.464 (3)	C16—C17	1.373 (3)
N6—C23	1.442 (3)	C16—H16A	0.9300
N6—C22	1.449 (3)	C17—C18	1.370 (3)
N6—C25	1.474 (3)	C17—H17A	0.9300
C2—C3	1.500 (3)	C18—C19	1.371 (3)
C3—C4	1.540 (3)	C18—H18A	0.9300
C3—C10	1.543 (3)	C20—H20A	0.9700
C3—C12	1.548 (3)	C20—H20B	0.9700
C4—C5	1.526 (3)	C21—C22	1.519 (3)
C4—H4A	0.9700	C21—H21A	0.9700
C4—H4B	0.9700	C21—H21B	0.9700
C5—C11	1.524 (3)	C22—H22A	0.9700
C5—C6	1.522 (3)	C22—H22B	0.9700
C5—H5A	0.9800	C23—C24	1.505 (3)
C6—C7	1.532 (3)	C23—H23A	0.9700
C6—H6A	0.9700	C23—H23B	0.9700
C6—H6B	0.9700	C24—H24A	0.9700
C7—C8	1.523 (3)	C24—H24B	0.9700
C7—C12	1.532 (3)	C25—C26	1.493 (4)
C7—H7A	0.9800	C25—H25A	0.9700
C8—C9	1.523 (3)	C25—H25B	0.9700
C8—H8A	0.9700	C26—H26A	0.9600
C8—H8B	0.9700	C26—H26B	0.9600
C9—C10	1.528 (3)	C26—H26C	0.9600
			
C1—N1—N2	113.17 (15)	C9—C11—H11B	109.9
C1—N1—C20	126.80 (16)	H11A—C11—H11B	108.3
N2—N1—C20	119.55 (15)	C7—C12—C3	109.61 (17)
C2—N2—N1	105.55 (14)	C7—C12—H12A	109.7
C1—N3—C2	109.05 (14)	C3—C12—H12A	109.7
C1—N3—N4	130.48 (15)	C7—C12—H12B	109.7
C2—N3—N4	120.39 (15)	C3—C12—H12B	109.7
C13—N4—N3	117.78 (16)	H12A—C12—H12B	108.2
C20—N5—C21	113.64 (16)	N4—C13—C14	121.94 (18)
C20—N5—C24	114.37 (17)	N4—C13—H13A	119.0
C21—N5—C24	110.13 (18)	C14—C13—H13A	119.0
C23—N6—C22	109.3 (2)	C19—C14—C15	113.81 (18)
C23—N6—C25	111.4 (2)	C19—C14—C13	126.64 (18)
C22—N6—C25	111.7 (2)	C15—C14—C13	119.52 (18)
N1—C1—N3	102.50 (15)	F1—C15—C16	118.7 (2)
N1—C1—S1	127.14 (14)	F1—C15—C14	116.88 (18)
N3—C1—S1	130.32 (14)	C16—C15—C14	124.4 (2)
N2—C2—N3	109.69 (16)	C15—C16—C17	118.3 (2)
N2—C2—C3	123.79 (16)	C15—C16—H16A	120.8
N3—C2—C3	126.41 (15)	C17—C16—H16A	120.8
C2—C3—C4	108.45 (15)	C18—C17—C16	120.7 (2)
C2—C3—C10	110.28 (14)	C18—C17—H17A	119.6
C4—C3—C10	108.29 (17)	C16—C17—H17A	119.6
C2—C3—C12	112.22 (16)	C17—C18—C19	118.8 (2)
C4—C3—C12	108.32 (15)	C17—C18—H18A	120.6
C10—C3—C12	109.18 (16)	C19—C18—H18A	120.6
C5—C4—C3	110.26 (17)	F2—C19—C18	117.7 (2)
C5—C4—H4A	109.6	F2—C19—C14	118.34 (17)
C3—C4—H4A	109.6	C18—C19—C14	123.9 (2)
C5—C4—H4B	109.6	N5—C20—N1	116.29 (16)
C3—C4—H4B	109.6	N5—C20—H20A	108.2
H4A—C4—H4B	108.1	N1—C20—H20A	108.2
C11—C5—C6	109.5 (2)	N5—C20—H20B	108.2
C11—C5—C4	109.72 (18)	N1—C20—H20B	108.2
C6—C5—C4	110.23 (19)	H20A—C20—H20B	107.4
C11—C5—H5A	109.1	N5—C21—C22	110.05 (18)
C6—C5—H5A	109.1	N5—C21—H21A	109.7
C4—C5—H5A	109.1	C22—C21—H21A	109.7
C5—C6—C7	108.82 (17)	N5—C21—H21B	109.7
C5—C6—H6A	109.9	C22—C21—H21B	109.7
C7—C6—H6A	109.9	H21A—C21—H21B	108.2
C5—C6—H6B	109.9	N6—C22—C21	110.9 (2)
C7—C6—H6B	109.9	N6—C22—H22A	109.5
H6A—C6—H6B	108.3	C21—C22—H22A	109.5
C8—C7—C6	109.7 (2)	N6—C22—H22B	109.5
C8—C7—C12	109.42 (17)	C21—C22—H22B	109.5
C6—C7—C12	109.98 (19)	H22A—C22—H22B	108.1
C8—C7—H7A	109.2	N6—C23—C24	110.55 (19)
C6—C7—H7A	109.2	N6—C23—H23A	109.5
C12—C7—H7A	109.2	C24—C23—H23A	109.5
C7—C8—C9	109.63 (18)	N6—C23—H23B	109.5
C7—C8—H8A	109.7	C24—C23—H23B	109.5
C9—C8—H8A	109.7	H23A—C23—H23B	108.1
C7—C8—H8B	109.7	N5—C24—C23	109.9 (2)
C9—C8—H8B	109.7	N5—C24—H24A	109.7
H8A—C8—H8B	108.2	C23—C24—H24A	109.7
C8—C9—C10	109.67 (19)	N5—C24—H24B	109.7
C8—C9—C11	109.57 (19)	C23—C24—H24B	109.7
C10—C9—C11	109.46 (19)	H24A—C24—H24B	108.2
C8—C9—H9A	109.4	N6—C25—C26	112.6 (2)
C10—C9—H9A	109.4	N6—C25—H25A	109.1
C11—C9—H9A	109.4	C26—C25—H25A	109.1
C9—C10—C3	109.92 (16)	N6—C25—H25B	109.1
C9—C10—H10A	109.7	C26—C25—H25B	109.1
C3—C10—H10A	109.7	H25A—C25—H25B	107.8
C9—C10—H10B	109.7	C25—C26—H26A	109.5
C3—C10—H10B	109.7	C25—C26—H26B	109.5
H10A—C10—H10B	108.2	H26A—C26—H26B	109.5
C5—C11—C9	109.09 (19)	C25—C26—H26C	109.5
C5—C11—H11A	109.9	H26A—C26—H26C	109.5
C9—C11—H11A	109.9	H26B—C26—H26C	109.5
C5—C11—H11B	109.9		
			
C1—N1—N2—C2	1.3 (2)	C6—C5—C11—C9	−60.9 (2)
C20—N1—N2—C2	173.83 (16)	C4—C5—C11—C9	60.2 (2)
C1—N3—N4—C13	−28.1 (3)	C8—C9—C11—C5	59.9 (2)
C2—N3—N4—C13	155.49 (19)	C10—C9—C11—C5	−60.4 (2)
N2—N1—C1—N3	−0.5 (2)	C8—C7—C12—C3	60.1 (2)
C20—N1—C1—N3	−172.39 (17)	C6—C7—C12—C3	−60.6 (2)
N2—N1—C1—S1	−178.51 (14)	C2—C3—C12—C7	178.87 (16)
C20—N1—C1—S1	9.6 (3)	C4—C3—C12—C7	59.2 (2)
C2—N3—C1—N1	−0.44 (19)	C10—C3—C12—C7	−58.5 (2)
N4—N3—C1—N1	−177.17 (17)	N3—N4—C13—C14	−179.08 (18)
C2—N3—C1—S1	177.48 (15)	N4—C13—C14—C19	2.1 (4)
N4—N3—C1—S1	0.7 (3)	N4—C13—C14—C15	179.9 (2)
N1—N2—C2—N3	−1.50 (19)	C19—C14—C15—F1	−178.7 (2)
N1—N2—C2—C3	−178.02 (16)	C13—C14—C15—F1	3.2 (3)
C1—N3—C2—N2	1.3 (2)	C19—C14—C15—C16	0.3 (3)
N4—N3—C2—N2	178.40 (15)	C13—C14—C15—C16	−177.7 (2)
C1—N3—C2—C3	177.68 (17)	F1—C15—C16—C17	178.8 (2)
N4—N3—C2—C3	−5.2 (3)	C14—C15—C16—C17	−0.2 (4)
N2—C2—C3—C4	−1.6 (2)	C15—C16—C17—C18	−0.4 (4)
N3—C2—C3—C4	−177.49 (17)	C16—C17—C18—C19	0.8 (4)
N2—C2—C3—C10	116.9 (2)	C17—C18—C19—F2	179.5 (2)
N3—C2—C3—C10	−59.1 (2)	C17—C18—C19—C14	−0.7 (4)
N2—C2—C3—C12	−121.18 (19)	C15—C14—C19—F2	179.9 (2)
N3—C2—C3—C12	62.9 (2)	C13—C14—C19—F2	−2.2 (4)
C2—C3—C4—C5	178.88 (16)	C15—C14—C19—C18	0.1 (3)
C10—C3—C4—C5	59.2 (2)	C13—C14—C19—C18	178.0 (2)
C12—C3—C4—C5	−59.1 (2)	C21—N5—C20—N1	59.6 (3)
C3—C4—C5—C11	−60.4 (2)	C24—N5—C20—N1	−68.1 (2)
C3—C4—C5—C6	60.3 (2)	C1—N1—C20—N5	−109.1 (2)
C11—C5—C6—C7	61.0 (2)	N2—N1—C20—N5	79.5 (2)
C4—C5—C6—C7	−59.8 (2)	C20—N5—C21—C22	173.51 (19)
C5—C6—C7—C8	−60.3 (2)	C24—N5—C21—C22	−56.7 (3)
C5—C6—C7—C12	60.1 (2)	C23—N6—C22—C21	−58.7 (3)
C6—C7—C8—C9	59.7 (2)	C25—N6—C22—C21	177.6 (2)
C12—C7—C8—C9	−61.1 (2)	N5—C21—C22—N6	57.6 (3)
C7—C8—C9—C10	60.8 (2)	C22—N6—C23—C24	59.7 (3)
C7—C8—C9—C11	−59.3 (2)	C25—N6—C23—C24	−176.4 (2)
C8—C9—C10—C3	−59.6 (2)	C20—N5—C24—C23	−172.79 (18)
C11—C9—C10—C3	60.6 (2)	C21—N5—C24—C23	57.8 (2)
C2—C3—C10—C9	−177.92 (17)	N6—C23—C24—N5	−59.5 (3)
C4—C3—C10—C9	−59.4 (2)	C23—N6—C25—C26	167.4 (3)
C12—C3—C10—C9	58.3 (2)	C22—N6—C25—C26	−70.1 (3)

### Hydrogen-bond geometry (Å, º)

Cg1 is the centroid of the N1–N3,C1,C2 ring

**Table:**

*D*—H···*A*	*D*—H	H···*A*	*D*···*A*	*D*—H···*A*
C18—H18*A*···N5^i^	0.93	2.58	3.451 (3)	157
C1—S1···*Cg*1^ii^	1.66 (1)	3.29 (1)	4.849 (2)	155 (1)

Symmetry codes: (i) *x*−1/2, −*y*+3/2, *z*−1/2; (ii) −*x*+3/2, *y*+1/2, −*z*+3/2.
